# Identification of sRNA mediated responses to nutrient depletion in *Burkholderia pseudomallei*

**DOI:** 10.1038/s41598-017-17356-4

**Published:** 2017-12-07

**Authors:** Hirzahida Mohd-Padil, Nadzirah Damiri, Suhaila Sulaiman, Shiao-Fei Chai, Sheila Nathan, Mohd Firdaus-Raih

**Affiliations:** 1 School of Biosciences and Biotechnology, Faculty of Science and Technology, Universiti Kebangsaan Malaysia, 43600 Bangi, Selangor Malaysia; 2 Malaysia Genome Institute, Jalan Bangi, 43600 Kajang, Selangor Malaysia; 3 FGV R&D Sdn. Bhd. Pt. 23417 Lengkuk Teknologi, 71760 Bandar Enstek, Negeri Sembilan Malaysia; 4 Institute of Systems Biology, Universiti Kebangsaan Malaysia, 43600 UKM Bangi, Selangor Malaysia

## Abstract

The *Burkholderia* genus includes many species that are known to survive in diverse environmental conditions including low nutrient environments. One species, *Burkholderia pseudomallei* is a versatile pathogen that can survive in a wide range of hosts and environmental conditions. In this study, we investigated how a nutrient depleted growth environment evokes sRNA mediated responses by *B*. *pseudomallei*. Computationally predicted *B*. *pseudomallei* D286 sRNAs were mapped to RNA-sequencing data for cultures grown under two conditions: (1) BHIB as a nutrient rich media reference environment and (2) M9 media as a nutrient depleted stress environment. The sRNAs were further selected to identify potentially *cis*-encoded systems by investigating their possible interactions with their flanking genes. The mappings of predicted sRNA genes and interactions analysis to their flanking genes identified 12 sRNA candidates that may possibly have *cis*-acting regulatory roles that are associated to a nutrient depleted growth environment. Our approach can be used for identifying novel sRNA genes and their possible role as *cis*-mediated regulatory systems.

## Introduction

The *Burkholderia* genus are generally soil bacteria that can invade a variety of host cells including human, animals and plants^1^. *Burkholderia* species, such as *Burkholderia pseudomallei*, *Burkholderia mallei* and *Burkholderia cepacia* are known to cause melioidoisis^2,3^, glanders^4^ and pulmonary infections in cystic fibrosis patients^5,6^, respectively. Others such as *Burkholderia plantarii*, *Burkholderia glumae* and *Burkholderia gladioli* have been reported to be pathogens of rice plant^7^. Several species such as *Burkholderia vietnamiensis*, *Burkholderia brasiliensis*, *Burkholderia phymatum* and *Burkholderia phytofirmans sp*. were reported to be beneficial to the environment and agriculture due to their involvement in atmospheric nitrogen fixation and plant growth stimulation^8–11^.

Despite having no endospore as a physical resistance feature, previous studies have reported that members of this genus are highly adaptable to various harsh and stress environments^12^. For example, *B*. *pseudomallei* is able to survive in distilled and rain water, with bacterial counts showing an increase of at least 2-fold in sterile distilled water and about 30-fold in rain water after a period of 28 days^13^. Other studies showed that *B*. *pseudomallei* can survive without any carbon source for several years^14^ as well as in hostile environments such as in detergent solutions^15^, in an acidic environment^16^ and in dehydrated soil with a water content of less than 10%^17^. The available literature have also reported the presence of post-transcriptional regulators comprising of non-coding RNAs (ncRNAs) that in addition to protein coding genes, can possibly contribute to *B*. *pseudomallei* versatility^18,19^. For example, Ooi and colleagues reported a total of 766 ncRNAs that were detected in the compendium of *B*. *pseudomallei* K96243 microarray expression profiles, whereby few examples of sRNA candidates such as *BPNC1007F* and *BPNC10061R* ncRNAs exhibited strong differential expression patterns due to nutrient deprivation treatment^18^. Other microarray analysis reported 38 novel sRNAs in *Burkholderia thailandensis*, of which 20 of them are also homologous to *B*. *pseudomallei* and *B*. *mallei* that exhibited differential expression in response to various stress treatments such as antibiotic challenges, anaerobic condition and exposure to hydrogen peroxide (H_2_O_2_)^20^.

Small RNAs (sRNAs) are highly structured functional transcripts that can form stem or hairpin loops and are commonly found in the intergenic regions with lengths varying from ~50–450 nucleotides^21^. sRNAs generally interact with target mRNA by complementarity base pairing interactions, either by blocking the ribosome binding site (RBS) and thus suppressing the translation of the mRNAs, or by unbinding the RBS to initiate the translation of target mRNAs^22^. Searching for sRNAs based solely on sequence similarity is hampered by the fact that many known homologous sRNAs tend to have poor sequence similarity and these sRNAs can be species-specific^23^. The mode of sRNA-mRNA interactions can be based on the location or the arrangement of the sRNAs, with respect to the loci of target mRNAs. For a *cis*-acting arrangement, the sRNA is located either in the 5′/3′ UTR of the mRNA^24,25^ or in the opposite strand to the target mRNA, whereby the latter is known as *cis* antisense sRNA^22^. Meanwhile, trans-acting sRNAs are encoded in a distant loci with respect to the target mRNA and these trans-acting sRNAs are known to be assisted by the RNA chaperone protein, Hfq^26,27^, to enhance the pairing interaction between sRNAs and the target mRNAs. An increasing number of functional sRNAs has been reported in diverse bacterial mechanisms, including biofilm formation^28^, quorum sensing^29^, virulence^30^ and stress response^31^. For example, RyhB and OxyS sRNAs in *Escherichia coli* were expressed during iron starvation and oxidative stress, respectively to avoid cell damage^32,33^. Meanwhile, another group of antisense sRNAs in *Pseudomonas aeruginosa* was reported to play a role in osmotic, oxidative and antibiotic stresses^34^.

In a previous study, Khoo *et al*.^19^ had developed a pipeline by integrating three sRNA prediction tools into a process to predict the sRNAs of *B*. *pseudomallei* K96243^35^ (Khoo_pipeline) and a small subset of the predictions were validated using reverse transcriptase polymerase chain reaction (RT-PCR). In this study, a search for sRNA homologs of *B*. *pseudomallei* K96243 in *B*. *pseudomallei* D286 were identified by re-mapping two independent datasets of putative sRNA candidates as previously predicted by Khoo_pipeline and from the work of Ooi *et al*.^18^ (Ooi_dataset). RNA-sequencing (RNA-seq) and sRNA-sequencing (sRNA-seq) data from *B*. *pseudomallei* D286 cultured in two different environmental conditions: nutrient rich brain heart infusion broth (BHIB) as a nutrient rich reference condition, and M9 minimal medium (M9) as a condition that simulates a nutrient depleted growth environment, were used to cross-reference and validate the expression of predicted sRNAs. The differential expression of *B*. *pseudomallei* D286 transcriptomes in a nutrient depleted environment revealed insights with regard to co-expression patterns between twelve validated sRNAs and their flanking genes. Added together to the potential *cis*-linkage between the sRNAs and their respective flanking genes, these pairings were amenable to further computational analysis for identifying putative RNA-RNA interactions, which could in turn be associated to potential mechanisms of *Burkholderia* sRNA mediated responses to low nutrient environments.

## Results

### Small RNA prediction in Bp_D286

Searches in the Bp_D286 genome using default parameters of Rfamscan identified 24 sRNA genes. We also identified 37 sRNAs in Bp_K96243 instead of Bp_D286 using sRNAscanner at default parameters because this program requires a *.ptt file that has been submitted to the Genbank database. The homologs for these 37 sRNAs were then identified and annotated in Bp_D286. The SIPHT program also required that a genome be already deposited in the Genbank database. Therefore we re-annotated the sRNA prediction in Bp_K96243 using SIPHT less stringently (by changing the E-value cutoff to 5e-3) to increase the coverage of our dataset and producing a search that yielded 1743 sRNA candidates in both chromosomes compared to the 1306 sRNAs as predicted by the Khoo_pipeline. We further added 766 sRNAs of Bp_K96243 from the Ooi_dataset into our analysis. The sRNAs from sRNAscanner, SIPHT and the Ooi_dataset were mapped against the Bp_D286 genome using BLASTN and redundant sRNA genes were merged into a combined total of 1822 sRNA genes - 1130 in the large chromosome and 692 in the small chromosome (Supplementary Table S1).

### Bp_D286 transcriptomes and differential expression analyses

In order to increase the coverage of our transcriptome, both RNA-seq and sRNA-seq samples were utilised. The standard RNA-seq protocol was carried out to detect both protein coding mRNAs and non-protein coding RNAs whereas the sRNA-seq method was intended to specifically detect bacterial sRNAs that are too small (~less than 200 bp) to be sequenced by standard RNA-seq. We were able to confirm the transcription of 1553 of the 1822 predicted sRNAs after cross-referencing with both RNA-seq and sRNA-seq data, respectively (Supplementary Table S2).

**Figure 1:**
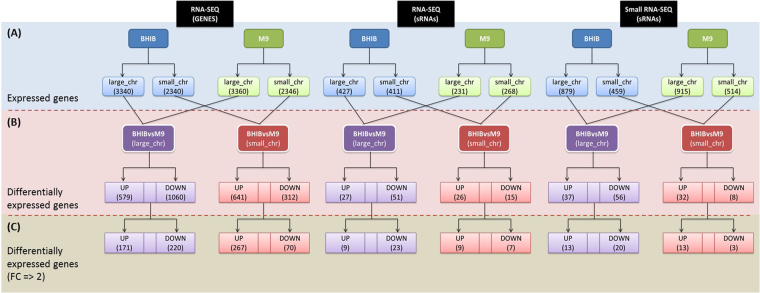
The number of expressed mRNAs and sRNAs from RNA-seq and sRNA-seq trancriptome assembly and those that are differentially expressed in M9 compared to the BHIB reference. (**A**) RNA-seq was used to obtain the list of mRNAs and sRNAs that are expressed in the nutrient rich (BHIB) and nutrient depleted (M9) cultures of *B*. *pseudomallei* D286 whereas the sRNA-seq method was used to identify expressed sRNAs in BHIB and M9. (**B**) The analyses for differential expression of mRNAs and sRNAs were performed by comparing the M9 minimal media against the BHIB reference condition. (**C**) Only mRNAs that are significantly differentially expressed were selected for *cis*-acting analysis.

After normalization of the replicates for the RNA-seq data; 3340 coding sequences (CDS) and 427 sRNAs in the large chromosome, and 2340 CDS and 411 sRNAs in the small chromosome were found to be transcribed when cultured in BHIB. In the M9 culture; 3360 CDS and 231 sRNAs in the large chromosome, and 2346 CDS and 268 sRNAs in the small chromosome were found to be expressed. For the sRNA-seq dataset; 879 sRNAs and 459 sRNAs were found to be expressed in the large and small chromosome respectively for BHIB sample while 915 sRNAs and 514 sRNAs were found to be expressed in the large and small chromosome respectively of M9 cultured samples (Fig. 1A).

The differential expression analyses between environmental conditions were performed using cuffdiff to compare the expression between the reference nutrient rich growth environment (BHIB) with the nutrient depleted M9 media. The annotated genes and sRNAs in genomic loci that do not have sufficient reads to be analyzed and those that did not pass the Benjamin Hochberg screening procedure using a false discovery rate (FDR) statistical approach were filtered out. In this statistical test, transcripts with q-values < 0.05 were considered significant experiment-wise. The differential expression analysis is based on the information of a fold change that is more than zero, in which the positive number (x > 0) showed that the genes/sRNAs were up-regulated whereas the negative number of fold change (x < 0) showed that the genes/sRNAs were down-regulated (Supplementary Table S3).

The differential expression analysis between BHIBvsM9 from RNA-seq data revealed 579 genes and 641 genes were up-regulated in the large and small chromosome respectively, whereas 1060 genes were down-regulated in the large chromosome and 312 genes were down-regulated in the small chromosome. For the same dataset, 27 sRNAs in the large chromosome and 26 sRNAs in the small chromosome were found to be up-regulated whereas 51 sRNAs in the large chromosome and 15 sRNAs in the small chromosome were found to be down-regulated. From the differential expression analysis in which we looked at the up-regulation or down-regulation of the sRNAs in M9 condition compared to BHIB growth from sRNA-seq data, 37 sRNAs were found to be up-regulated and 56 sRNAs were down-regulated in large chromosome whereas in small chromosome, 32 and eight sRNAs were found to be up- and down-regulated, respectively (Supplementary Table S4) (Fig. 1B).

### The prediction of sRNA based on associated genes and transcriptome expression

In order to find potential sRNAs in *Burkholderia* species that respond to nutrient depleted environment, we inspected the differential expression of the sRNA and their flanking genes by first collecting the pattern of co-expression between detected sRNAs and their adjacent genes.

Based on the differential expression between M9 and BHIB reference, we then further selected the co-expressed sRNAs and their genes with the fold change (FC) equal to or more than |2|. This filter, when implemented on the RNA-seq dataset, identified 17 CDS and nine sRNAs that were significantly up-regulated and 220 CDS and 23 sRNAs that were down-regulated in the large chromosome. The same filter when applied to the sRNA-seq dataset of the small chromosome identified 267 CDS and nine sRNAs that were significantly up-regulated and 70 CDS and seven sRNAs that were significantly down-regulated. For the sRNA-seq dataset, 13 sRNAs were significantly up-regulated in each chromosome, while 20 sRNAs in the large chromosome and three sRNAs in the small chromosome were down-regulated. As a result, a total of 47 pairings exhibited a physical co-linkage between sRNAs and the genes (Supplementary Table S5) (Fig. 1C).

*B*. *pseudomallei* K96243 homologs were used to represent genes in the D286 strain, we computed the possible interactions of these 47 sRNAs with their flanking genes using CopraRNA^36^. This representation using the K96243 genome was necessary because CopraRNA are only able to analyze bacterial genomes in Refseq or Genbank. From the 47 *cis*-acting candidates, 12 sRNAs were predicted to interact with their flanking genes significantly, hence suggesting their potential as *cis*-linked sRNAs. Other than the immediate adjacent gene, distantly encoded target genes were also predicted by CopraRNA (Supplementary Table S6). Of these, nine sRNAs were located at 5′ UTR of their putative target mRNAs (BPNC10044R, BPNC10113R, BPNC10048R, BPNC10196F_1129_SIPHT, BPNC10209R, BPNC10146R, BPNC10233R_28_SIPHT, BPNC10134F and BPNC10146F). The other sRNAs, Candidate_369_SIPHT and Candidate_468_SIPHT were encoded within the opposite strand to the target mRNAs, whereas BPNC10037R was predicted to act upon two different genes that are located within both 5′ UTR and antisense strand of the two target mRNAs.

**Figure 2:**
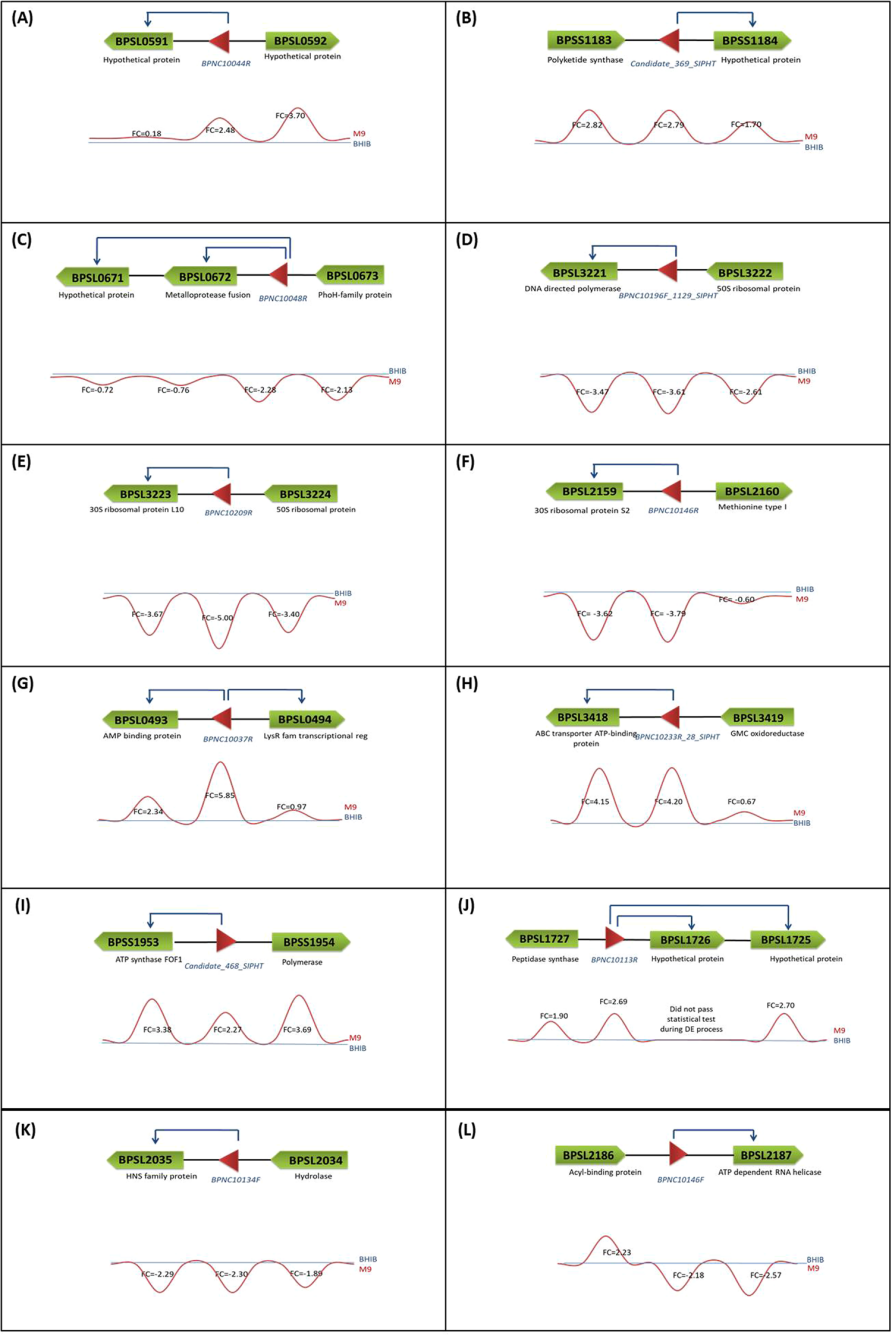
A total of 12 out of 58 *B*. *pseudomallei* D286 sRNAs that are predicted to be *cis*-acting regulators based on CopraRNA prediction. (**A**–**C**) Show sRNAs that are predicted to interact with mRNAs that were not significantly differentially expressed. (**D**–**F**) Show sRNAs for which the mRNAs on both flanks were down-regulated. These pairings (*bpsl3221*:BPNC10196F_1129_SIPHT, *bpsl3223*:BPNC10209R, *bpsl2159*:BPNC10146R) were in a co-linear arrangement that contain the ribosomal proteins. (**G**) BPNC10037R is predicted to interact the mRNAs from its 3′ as well as 5′ side. (**H**–**L**) Show sRNAs that is predicted to interact with the mRNAs that were significantly differently expressed, either up-regulated or down-regulated.

Of the 15 flanking genes that were found to interact with these 12 sRNAs, seven genes were not significantly expressed based on their FC value. BPNC10044R was predicted to interact with *bpsl0591*, a hypothetical protein (FC = 0.18) (Fig. 2A); Candidate_369_SIPHT that was predicted to interact with *bpss1184*, also a hypothetical protein (FC = 1.70) (Fig. 2B); and BPNC10048R that was predicted to interact with two genes at its 3′ site - *bpsl0672*, a metalloprotease fusion protein (FC = 0.76) and *bpsl0671*, a hypothetical protein (FC = 0.72) (Fig. 2C). Three sRNA candidates, BPNC10196F_1129_SIPHT, BPNC10209R and BPNC10146R, are located between genes encoding ribosomal proteins. All of these sRNAs and their flanking genes (*bpsl3221*, *bpsl3222*, *bpsl3223*, *bpsl3224*, *bpsl2159 and bpsl2159b*) were down-regulated (Fig. 2D–F).

BPNC10037R was significantly up-regulated (FC = 5.8) in M9 culture and interestingly, it was predicted to have possible interactions with flanking genes on both the 5′ and 3′ end - *bpsl*0494 (LysR family transcriptional regulator; FC = 0.97) and *bpsl*0*493* (AMP binding protein; FC = 2.34) (Fig. 2G). The up-regulated (FC = 4.20) BPNC10233R_28_SIPHT was predicted to interact with its adjacent gene, *bpsl3418* (ABC transporter ATP binding protein; FC = 4.15) (Fig. 2H). Candidate_468_SIPHT was another sRNA that was predicted to interact with the gene located on its 5′ side, *bpss1953* (an ATP synthase FOF1; FC = 3.38) (Fig. 2I). BPNC10113R was predicted to interact with *bpsl1725*, a hypothetical protein (FC = 1.90) (Fig. 2J).

BPNC10134F was predicted to interact with the mRNA on its 5′ side that encoded for *bpsl2035*, a regulatory protein containing H-NS histone family domain - both were down-regulated in the M9 culture (Fig. 2K). The other sRNA-mRNA interaction that was adjacent to each other and also down-regulated in the M9 culture was BPNC10146F that flanked *bpsl2187* (an ATP dependent RNA helicase; FC = −2.57). In contrast to the 11 other *cis*-acting sRNA candidates where their 5′ gene and 3′ gene were either up- or down-regulated together, this is the only case in which the 3′ end gene and 5′ end gene had different expression patterns (Fig. 2L).

### Small RNA conservation

From the conservation analysis of these 12 *cis*-acting sRNA candidates based on sequence and computed structural similarities using the BLAST and Infernal programs respectively, 11 were conserved only in *Burkholderia* species. When structure based conservation analysis was done using Infernal, another *cis*-acting sRNA candidate, BPNC10196F_1129_SIPHT that is conserved in many *Burkholderia* species, also has homologs in two other species of the *Burkholderiales* order, *Verminephrobacter eiseniae* EF01-2 and *Leptothrix cholodnii* SP-6. BPNC10146F, BPNC10209R and BPNC10233R_28_SIPHT were found in many *Burkholderia* species while BPNC10037R, BPNC10048R and BPNC10113R were consistently found uniquely in *B*. *pseudomallei* and *B*. *mallei* by both methods used to identify sRNA homologs. One *cis*-encoded sRNA candidate, Candidate_369_SIPHT was found only in *B*. *pseudomallei* and *B*. *thailandensis*, while BPNC10044R, BPNC10134F, BPNC10146R and Candidate_468_SIPHT were found to be conserved in *B*. *pseudomallei*, *B*. *mallei* and *B*. *thailandensis*. All the conservation analyses were also extended to the homologous flanking genes of the homologous sRNAs.

### Validation of relative expression of both sRNA candidates and flanking genes using reverse transcription PCR (RT-PCR) and quantitative PCR (qPCR) assays

To substantiate whether similar RNA-seq-derived expression pattern of 12 *cis*-acting sRNA candidates including their flanking genes in Bp*_*D286 could be observed during rapid initiation growth of the bacterial strain at its mid-log phase, these candidates were further subjected to both semiquantative RT-PCR and qPCR assays. The result of experimental validations showed that 10 out of 12 sRNA:flanking gene pairings were consistently amplified using the designed primers (Supplementary Table S7 and Supplementary Figure 8), demonstrating the reproducibility of both putative sRNA and the gene expression that were initially revealed by RNA-seq data (Table 1). However, the amplification of other two sRNA candidates, Candidate_369_SIPHT and Candidate_468_SIPHT were not successful. If these sRNA candidates were truly transcribed under the given conditions, it was presumed that the usage of random hexamers as a standard practice to generate first-strand cDNAs might hamper the priming of the three sRNA candidates due to their high GC content of more than 63%^37^.

**Table 1:** Summary of expression patterns between sRNA and its flanking gene(s) based on differential expression using RNA-seq and qRT-PCR analyses. Using RNA-seq and qRT-PCR analyses, the differential expression of both sRNA and its flanking gene(s) in Bp D286 under M9 growth condition in comparison to BHIB (reference condition) was summarised in the table using the designation of expression pattern as: (i) ‘up’ = up-regulated, (ii) ‘down’ = down-regulated, (iii) ‘nd’ = not detected, or (iv) ‘na’ = not available due to non-detection of upstream sRNA. The grey shades represent the congruency in terms of expression pattern of sRNA:flanking gene pairings between the two column analyses.

*cis*-sRNA	Locus tag of target gene(s)	RNA-seq		qRT-PCR	
		sRNA	gene(s)	sRNA	gene(s)
BPNC10044R	*bpsl0591*	up	up	up	up
BPNC10113R	*bpsl1725*	up	up	up	up
Candidate_369_SIPHT	*bpss1184*	up	up	nd	na
BPNC10048R	*bpsl0671*, *bpsl0672*	down	down, down	up	nd, up
BPNC10196F_1129_SIPHT	*bpsl3221*	down	down	up	down
BPNC10209R	*bpsl3223*	down	down	down	down
BPNC10146R	*bpsl2159*	down	down	down	down
BPNC10037R	*bpsl0493*, *bpsl0494*	up	up, up	up	up, up
BPNC10233R_28_SIPHT	*bpsl3418*	up	up	up	up
Candidate_468_SIPHT	*bpss1953*	up	up	nd	na
BPNC10134F	*bpsl2035*	down	down	down	down
BPNC10146F	*bpsl2187*	down	down	up	up

**Figure 3:**
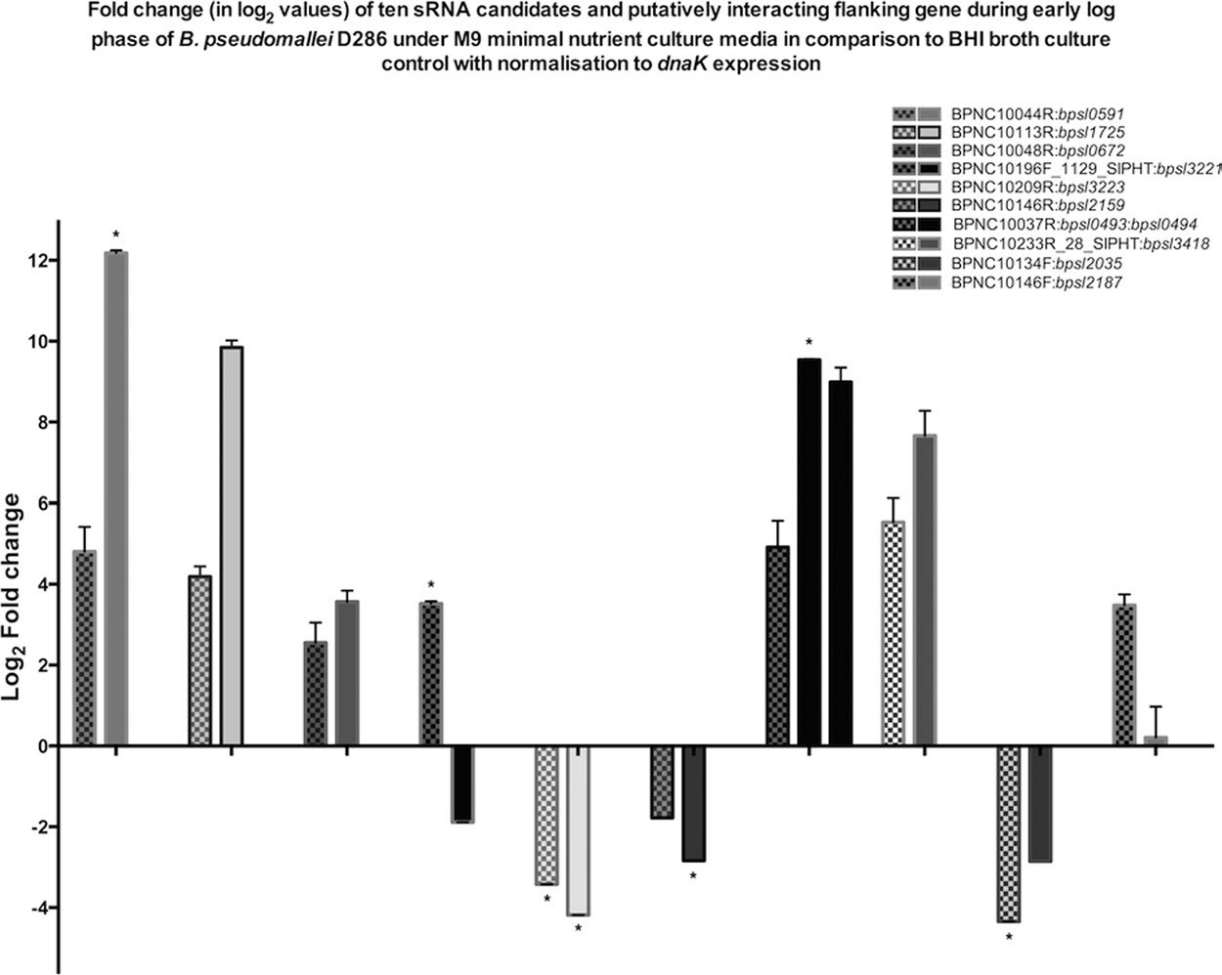
Relative expression (in fold change value) of eleven PCR-validated sRNA candidates with respective flanking genes as determined by qRT-PCR. The differential expression (in fold change) of PCR-validated sRNA:flanking gene pairings for *B*. *pseudomallei* D286 under minimal nutrient treatment in comparison to the nutrient rich culture reference (fold change_M9/BHIB_). For each pair, the checquered bars represent the fold change_M9/BHIB_ (log2-transformed) of putative sRNA expression, whereas the plain bars represent the fold change_M9/BHIB_ (log2-transformed) of putatively-interacting flanking gene expression. The relative expression (mean ± sem) of each sRNA candidate and flanking gene were normalised to *dnaK* using ΔΔCT method. Using statistical one sample *t*-test (H_*0*_ = 1), any fold change of sRNA or gene expression that scored *p*-value of <0.05 is indicated with asterisk (*).

The transcriptional effects among seven sRNA:flanking gene pairings were concordantly observed between RNA-seq and qPCR data, as either being up-regulated (BPNC10044R:*bpsl0591*, BPNC10113R:*bpsl1725*, BPNC10037R:*bpsl0493*-*0494*, BPNC10233R_28_SIPHT:*bpsl3418*) or down-regulated (BPNC10209R:*bpsl3223*, BPNC10146R:*bpsl2159* and BPNC10134F:*bpsl2035*) upon M9 treatment compared to the reference expression under nutrient-rich BHIB (Table 1). The differential expressions of BPNC10048R:*bpsl0672*, BPNC10146F:*bpsl2187* and BPNC10196F_1129_SIPHT sRNA (except its flanking gene, *bpsl3221*) exhibited changes but in opposite directions upon the nutrient-limited treatment towards Bp*_*D286. The following relative expression levels of sRNA/flanking gene were presented in log_2_-transformed values. The expression ratio of BPNC10048R:*bpsl0672*, BPNC10146F:*bpsl2187* and BPNC10196F_1129_SIPHT sRNA was moderately up-regulated (≤3.6–fold increase) as compared to the concordant pairs of sRNA:flanking gene, which were up-regulated by more than 4–fold (Fig. 3). On the contrary, the down-expression of three concordant pairs as well as *bpsl3221* exhibited a reduction by more than 1.8-fold in response to minimal-nutrient treatment.

## Discussion

In this study, we report the expression profiles of twelve predicted *cis*-encoded sRNAs that were differentially expressed in *B*. *pseudomallei* D286 upon nutrient-stress treatment. Based on the comparative transcriptomic analysis between two standard laboratory conditions (BHIBvsM9), the nutrient-stress induction of *B*. *pseudomallei* D286 triggered a parallel differential expression among six sRNA candidates towards the direction of being up-regulated as compared to their reference expression under BHIB condition (Fig. 3, Table 1). It is noted that the subtle increase in these sRNA expression levels correlates with transcriptional burst of their flanking genes as well. Among these co-expressed sRNA:flanking gene pairings, BPNC10037R:*bpsl0493* and BPNC10113R:*bpsl1725* were previously reported to be associated with respective gene clusters of small molecule metabolic pathway and amino acid biosynthesis in *B*. *pseudomallei* K96243^18^. It is presumed that these sRNA expression profiles may reflect an interplay of regulatory consequence exerted by these putative sRNAs in order to accommodate bacterial adaptation to nutrient depletion *in vitro*. Besides, the differential expressions of the up-regulated sRNAs are shown to be subtle under nutrient-stress condition, which reflect a narrow shift in terms of their steady-state level between the two nutrient treatments. Perhaps these constitutively-expressed sRNAs act like a dial, allowing them to selectively tune the gene expressions that are required upon a sudden change in nutrient availability^38^. Hence, the co-expression of potentially *cis*-linked sRNAs and flanking genes in *B*. *pseudomallei* D286 suggests a likelihood for their transcriptional regulation to support bacterial adaptation in nutrient stress collectively^39^.

The BPNC10146R, BPNC10209R and BPNC10134F sRNA candidates were observed to be co-downregulated together with their target mRNAs, *bpsl2159* (30S ribosomal protein S2), *bpsl3223* (30S ribosomal protein L10) and *bpsl2035* (H-NS protein) respectively. Among these sRNA-flanking gene pairings, it is striking to observe a parallel downregulation of these sRNAs, which are concomitantly linked to genes encoding proteins that negatively control one’s own mRNA abundance^40,41^. Hence, it is tempting to propose that perhaps the autoregulation of ribosomal protein S2 and L10 subunits as well as *hns* transcription may require these sRNAs as potential mediators against their own expression. Two independent studies have reported compelling findings of twelve structured RNA elements as well as sRNA t44 (RFAM: RF00127) that are linked to S2 and L10 subunit genes in *E*. *coli* ^42,43^.

Only a handful of similar aforementioned findings have been reported in other bacteria, including SAR11 marine bacteria, ‘*Candidatus* pelagibacter ubique’ and *Bordetella pertussis* ^44,45^. Nevertheless, such apparent down-expression of the ribosomal subunit genes shown in this study is suggested to be linked with strong specific repression of BPNC10209R and BPNC10134F sRNAs, both of which may indirectly involve a far more complex mechanism that eventually forfeits the availability of cognate ribosomal RNAs. Likewise, the co-down-expression of BPNC10134F:*bpsl2035* in this study may also be explained by strong repression of BPNC10134F sRNA, which possibly confer negative regulation against *hns* transcription. Other similar *cis*-linkage between computationally predicted Arrc03 sRNA and *hns* gene has been previously reported in *Actinobacillus pleuropneumoniae* ^46^. Taken together, such tandem linkages between putative sRNAs and genes wherein the autoregulatory expression is extended to strongly suggest that the downregulation of the potentially *cis*-linked sRNAs of *B*. *pseudomallei* D286 may indirectly involve in a negative feedback mechanism of ribosomal subunit proteins and also biosynthesis of global transcription repressor, H-NS protein under nutrient-stress condition.

To further interrogate whether the *cis*-linked sRNA candidates were likely to form direct base-pairing with their cognate mRNA, the CopraRNA analyses predicted that the following sRNAs, BPNC10113R, BPNC10037R and BPNC10233R_28_SIPHT might readily form duplexes with their respective flanking mRNAs via nearly perfect ~100 bp interaction (Supplementary Table S9). Apart from the apparent co-expression between the sRNA candidates and their flanking genes, CopraRNA analysis also enlisted other distantly encoded target mRNAs of these selected sRNAs, which offers another possibility for these sRNAs to putatively interact with other mRNA targets. Added together with the conservation of *cis*-linkage between sRNAs and their flanking genes within *Burkholderia* species, it is suggested that rather than a straight-forward interaction due to their immediate vicinity, perhaps a more complex association and cross-talk of hierarchical targeting by any of these sRNAs lead to the downstream transcriptional change of their respective flanking genes^47^.

While there are advantages to be gained by using computational pipelines for genome-wide sRNA discovery in Bp_D286, it is worth noting that *in silico*-predicted sRNA candidates in the initial screening largely succumbed to a high rate of false positive results^48^. By combining both datasets of computational sRNA prediction and RNA-seq/sRNA-seq data, this approach is proven to further condense the list of *B*. *pseudomallei* sRNAs that potentially operate as RNA regulators under specific comparisons in response to nutrient depletion. Such congruent agreement of sRNA:flanking gene co-expression between RNA-seq and qPCR data analyses in this study demonstrates the utility of RNA-seq data that even without the satisfactory depth can be used as a discovery platform, which can be validated by less costly means such as qRT-PCR.

The fact that the distribution of sRNA homologs reported in this study is confined particularly within *Burkholderia* genus, none of these *cis*-linked sRNA candidates are currently supported by orthologs to known *cis*-acting sRNAs involved in nutrient stress response. Other related findings of putative sRNA identification have been previously described in other *B*. *pseudomallei* K96243 strain^18,19^. Among 766 ncRNAs that reported in the compendium study of the strain aforementioned, *BPNC10061R* ncRNA was shown to exhibit strong differential expression pattern due to the treatment of nutrient deprivation and its homologous sequences were detected in *B*. *mallei*, *B*. *thailandensis* and *B*. *cenocepacia* ^18^. This raises the question of whether these genus-specific sRNA candidates are key components of survival strategies for *Burkholderia* sp., such as *B*. *mallei*, *B*. *thailandensis* and *B*. *pseudomallei* to persist and adapt to diverse environments and specifically for this study, those that have limited nutrients.

In summary, most studies on pathogens such as *B*. *pseudomallei* have focused on the virulence and pathogenesis aspects that can lead to infection and disease. Nevertheless, for pathogens such as *B*. *pseudomallei*, their capacity to contaminate and persist in low nutrient and stressful environments such as water reservoirs is of epidemiological concern. Our study has implicated a significant role being played by sRNAs in responding and regulating survival mechanisms for a nutrient depleted environment. Among the sRNAs identified include a potentially novel sRNA gene. This knowledge may in the future be compounded to other studies in order to understand survival mechanisms in nutrient depleted environments thus paving the way for future strategies to manage potential contamination and latent survival issues.

## Methods

### Small RNA predictions and annotations

**Figure 4:**
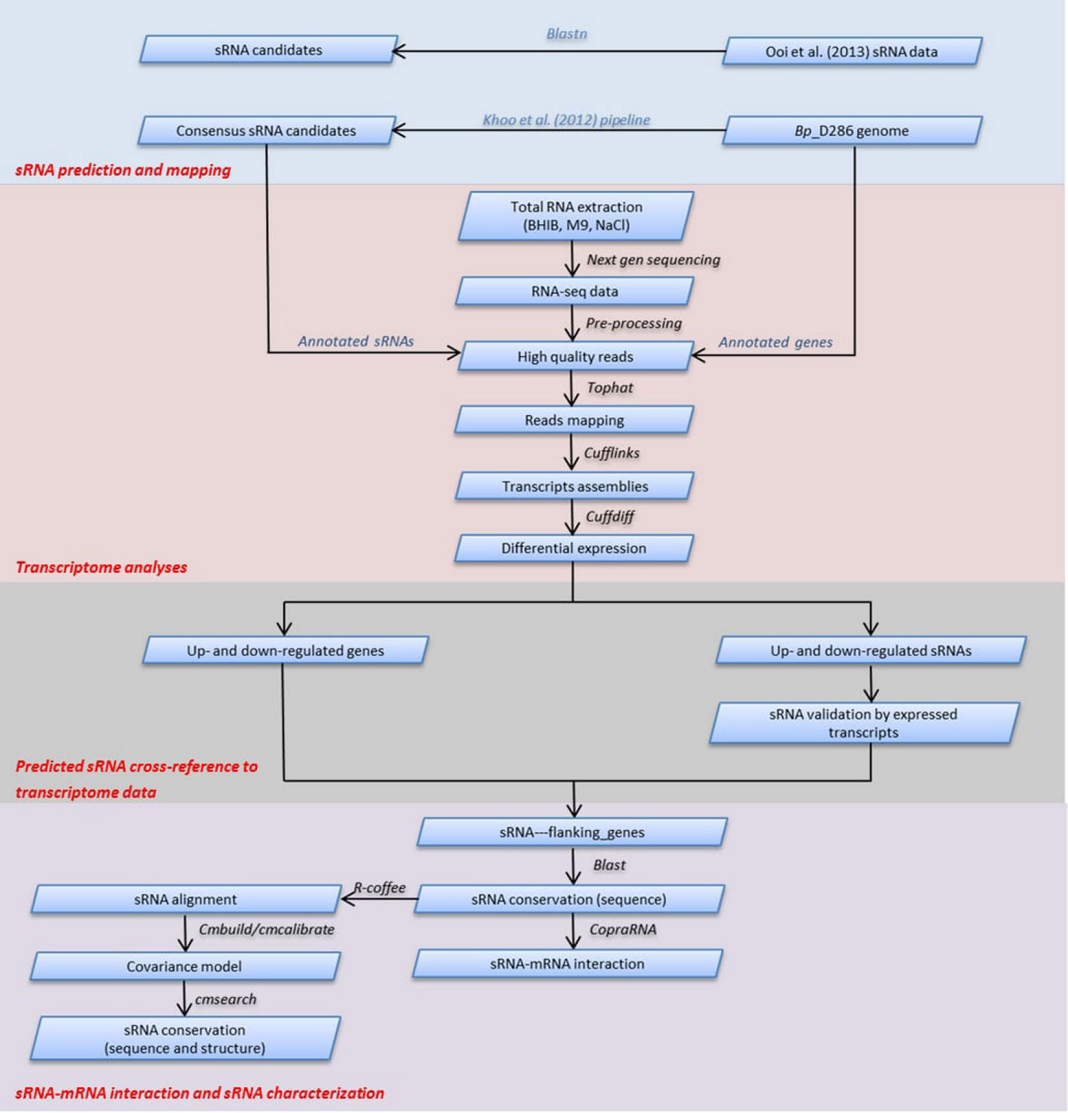
The pipeline for sRNA prediction, sRNA validation by transcriptome cross-referencing, sRNA-mRNA interaction analyses and sRNA conservation based on sequence and structure similarities. (**A**) The dataset for sRNAs was collected from Ooi *et al*.^18^ and by using a modified version of the method reported by Khoo *et al*.^19^; (**B**) RNA-seq data from B. pseudomallei grown in different culture media were processed for differential expression analysis; (**C**) These sRNAs then were cross-referenced with RNA-seq data to validate their expressions; (**D**) For sRNAs that were observed to have flanking genes with significant differential expression, the interaction between sRNA-mRNA were analyzed using copraRNA followed by conservation analysis based on both sequence and secondary-structural similarities.

The Khoo_pipeline that integrated three individual sRNA prediction software: SIPHT^49^, Rfamscan/Infernal^50^ and sRNAscanner^51^ was used to predict sRNA genes in the *B*. *pseudomallei* D286 (Bp_D286) genome which had been sequenced using single molecule real-time sequencing platform (Pacific Biosciences). Additionally, sRNAs from the Ooi_dataset for Bp_K96243 was also used to identify their Bp_D286 counterparts and for cross-referencing with transcriptome data from RNA-sequencing (Fig. 4).

### *B*. *pseudomallei* strain and growth conditions

*B*. *pseudomallei* D286, a clinical isolate previously described by Lee *et al*.^52^, was streaked on standardly-prepared Ashdown agar plate and incubated at 37 °C for approximately 48 hours. Single colonies of the isolate were inoculated into BHIB and were shake-cultured overnight at 37 °C. For preparation of RNA-seq and sRNA-seq samples, the overnight seed cultures were subcultured into fresh BHIB and continued to shake until the O.D_600_ reached a value of ~0.4–0.6. Two technical replicates of each subculture pool were diluted at 1:1 into either fresh BHIB or M9 minimal medium. After an hour of shaking at 37 °C, each cell suspension of both BHIB- and M9-dilution was pelleted and the decanted cells were homogenized with ~3 mL of TRIzol LS Reagent® (Invitrogen), and stored at −80 °C until further use for RNA extraction.

### RNA extraction and transcriptome sequencing

Total RNA samples of *B*. *pseudomallei* D286 were extracted according to the standard Trizol protocols. Briefly, the TRIzol-homogenized cells were suspended with chloroform and centrifuged to separate the phases. The RNA contained in the aqueous phase were precipitated using isopropyl alcohol and the RNA pellets were washed with 75% ethanol and further suspended in 30 μL of DEPC-treated water. The RNA samples were subjected to sequential precipitation with 3 M of sodium acetate washing and the samples were pelleted by cool centrifugation at 12,000 rpm. The RNA pellets were further washed with 75% ethanol and the decanted RNA pellets were resuspended with 30 μL of DEPC-treated water and treated with DNase I as to eliminate any possibility of genomic contamination. For the transcriptome sequencing RNA samples, the replicates were designated according to the culture-induced media as follows: for BHIB-induced, T1 and T2 for RNA-seq; T7 and T8 for sRNA-seq, whereas for M9-induced, T5 and T6 for RNA-seq; T11 and T12 for sRNA-seq. All prepared samples with each approximately 50 μg of total RNA concentration were sent to BGI, Shenzen, China for transcriptome sequencing on the Illumina HiSeq 2000 platform.

### Transcriptome analyses

After transcriptome sequencing, the RNA-seq datasets were pre-processed by trimming out reads with low base quality (Qv = 20) and short reads less than 50 nt. For the sRNA-seq data, pre-processing were carried out by trimming the low quality reads, filtering for 5′ primer contaminants, removing poly A reads and removing reads that were shorter than 18 bp. Taking the annotation information of genes and sRNAs, read mapping and transcriptome assembly for each sample was performed using Tophat and cufflinks^53^ with the *B*. *pseudomallei* D286 genome as a reference. Cuffmerge was used to merge the assembly datasets from all transcriptome conditions. Differential expression analysis of sRNAs between the BHIB control condition versus nutrient-depleted M9 condition (BHIBvsM9) was performed using the cuffdiff program. In this analysis, both replicates for each sample were normalized and the differential expression for each gene and sRNA were measured using fold-change value that was formulated based on log2 (FPKMy/FPKMx).

### sRNA function predictions based on associated genes and transcriptome expression

For selected candidates, the pattern(s) of UP and DOWN for the sRNA genes and their associated flanking genes were observed in order to identify potential *cis*-acting mechanisms. The computer programs CopraRNA and IntaRNA were used to predict sRNA-mRNA interactions^36^. sRNAs which had their flanking genes predicted to be their target mRNAs were further analyzed in terms of their RNA motif structures and conservation based on sequence and secondary structures. BLASTN^54^ analysis was performed against our in-house sRNA dataset predicted from 2470 complete bacterial genomes from Genbank; sRNAs with zero match against the in-house dataset were further searched against non-redundant nucleotide databases to find any other potential homologs. The alignment of the sequences obtained from BLAST was built using R-coffee^55^ and the consensus secondary structure was built using RNAalifold^56^ integrated in the T-coffee program. The covariance model (cm model) representing the sRNA alignment was built and calibrated using cmbuild and cmcalibrate from the Infernal package^50^. An extensive search of the sRNA homologs against other sequences from the bacterial Kingdom present in the Genbank database was performed using cmsearch in Infernal. The search was performed by implementing the covariance model constructed as input with the cut-off value of 1e-5. The ‘closest-features’ option in the BEDOP program was used to obtain the flanking genes in order to confirm the flanking genes of all hits were homologous to flanking genes of the query sRNA^57^. Cmalign from the Infernal package was later used to align the chosen sRNA sequences obtained from cmsearch analysis.

### Real-time quantitative PCR

Approximately 800 ng of each input total RNA were reverse transcribed into cDNAs using Superscript III reverse transcriptase (Invitrogen) and random hexamers as described by the manufacturer’s protocol. About 40 ng of cDNAs was used as template for relative quantification of different targets using qPCR amplification assay. The primer sequences of each target were provided in Supplementary Table 7. All qPCR reactions were performed on the CFX96 thermal cycler (Bio-Rad) using qPCRBIO SyGreen Mix Lo-ROX (PCR Biosystem) with thermocycling conditions as per the instructions given in the manual. The relative expression level of each sRNA/flanking gene was normalized against *dnak* mRNA expression (mean ± SEM; n = 4). The fold change of each target expression was both calculated based on 2^−ΔΔCT^ method and statistically analysed using one sample *t*-test, following the treatment of *B*. *pseudomallei* D286 under minimal nutrient M9 culture medium as compared to BHIB culture condition.

## Electronic supplementary material

Supplementary information

Supplementary_Table_S1

Supplementary_Table_S2

Supplementary_Table_S3

Supplementary_Table_S4

Supplementary_Table_S6

Supplementary_Table_S9
