# Supplementary material for: Identification of sRNA mediated responses to nutrient depletion in Burkholderia pseudomallei
Source: Sci Rep. 2017 Dec 7;7:17173. doi: 10.1038/s41598-017-17356-4 (PMC5719362; doi:10.1038/s41598-017-17356-4)
Supplement: Supplementary file 1 — Supplementary information [file 41598_2017_17356_MOESM1_ESM.pdf]

SUPPLEMENTARY INFORMATION

Identification of sRNA mediated responses to nutrient depletion in *Burkholderia pseudomallei*

Hirzahida Mohd-Padil[1, 2], Nadzirah Damiri[1], Suhaila Sulaiman[1, 3], Shiao-Fei Chai[1], Sheila Nathan[1], Mohd Firdaus-Raih[1, 4,*]

[1]School of Biosciences and Biotechnology, Faculty of Science and Technology, Universiti Kebangsaan Malaysia, 43600 Bangi, Selangor, Malaysia
[2]Malaysia Genome Institute, Jalan Bangi 43600 Kajang, Selangor, Malaysia
[3]FGV R&D Sdn. Bhd. Pt. 23417 Lengkuk Teknologi, 71760 Bandar Enstek, Negeri Sembilan, Malaysia
[4]Institute of Systems Biology, Universiti Kebangsaan Malaysia, 43600 UKM Bangi, Selangor, Malaysia

***Contact information: firdaus@mfrlab.org**

Supplementary Information

Supplementary Table S1 – Details of sRNAs predicted for the Bp_D286 genome sequence using several different programs

*Provided in Supplementary_Table_S1.xls

Sheet 1: (A) D286_rfamscan
Identification of sRNAs in Bp_D286 using Rfamscan.

Sheet 2: (B) K96243_srnascanner
Identification of sRNAs in Bp_K96243 using sRNAscanner.

Sheet 3: (C) K96243_SIPHT_chrL
Identification of sRNAs in large chromosome of Bp_K96243 using SIPHT.

Sheet 4: (D) K96243_SIPHT_chrS
Identification of sRNAs in small chromosome of Bp_K96243 using SIPHT.

Sheet 4: (E) K96243_Ooi_chrL
sRNAs predicted by Ooi et al. (2013) in large chromosome of Bp_K96243.

Sheet 5: (F) K96243_Ooi_chrS
sRNAs predicted by Ooi et al. (2013) in small chromosome of Bp_K96243.

Sheet 6: (G) D286_predicted_srna_chrL
Final sRNAs prediction in large chromosome of Bp_D286 using all integrated programs.

Sheet 7: (H) D286_predicted_srna_chrS
Final sRNAs prediction in small chromosome of Bp_D286 using all integrated programs.

Supplementary Table S2 – sRNAs in Bp_D286 validated via transcriptome (sRNA/RNA-seq) cross-referencing

*Provided in Supplementary_Table_S2.xls

Sheet 1: (A) Validated_expressed_chrL
sRNAs validation in large chromosome of Bp_D286 via transcriptome cross-referencing.

Sheet 2: (B) Validated_expressed_chrS
sRNAs validation in small chromosome of Bp_D286 via transcriptome cross-referencing.

Supplementary Table S3 – The differential expression results of annotated genes in Bp_D286 between BHIB RNA-seq and M9 RNA-seq conditions

*Provided in Supplementary_Table_S3.xls

Sheet 1: (A) CDS_bhibvsm9_chrL
The differential gene expression results between BHIB and M9 condition in large chromosome of Bp_D286 (RNA-seq data).

Sheet 2: (B) CDS_bhibmsm9_chrS
The differential gene expression results between BHIB and M9 condition in small chromosome of Bp_D286 (sRNA-seq data).

Supplementary Table S4 – The differential expressions results of predicted sRNAs in Bp_D286 between BHIB and M9 conditions (both RNA-seq and sRNA-seq data)

*Provided in Supplementary_Table_S4.xls

Sheet 1: (A) bhibvsm9_chrL_rnaseq
The differential sRNA expression results between BHIB and M9 condition in large chromosome of Bp_D286 (RNA-seq data).

Sheet 2: (B) bhibvsm9_chrS_rnaseq

The differential sRNA expression results between BHIB and M9 condition in small chromosome of Bp_D286 (RNA-seq data).

Sheet 3: (C) bhibvsm9_chrL_srnaseq

The differential sRNA expression results between BHIB and M9 condition in large chromosome of Bp_D286 (sRNA-seq data).

Sheet 4: (D) bhibvsm9_chrS_srnaseq

The differential sRNA expression results between BHIB and M9 condition in small chromosome of Bp_D286 (sRNA-seq data).

Supplementary Table S5 – A pattern of expressions of the sRNAs and their flanking genes that possibly interact via *cis*-acting mechanism in BHIBvsM9 condition

No	Data	Chr	sRNAs	sRNA ID	SRNA start	sRNA stop	Flanking genes	Gene ID	K96243 homolog	Descriptions
1	RNA-seq	chrL	BPNC10037R	D286.1_srna_0144	510871	511130	Amp binding protein	D286.1_0466	BPSL0493	Gene UP and sRNA UP in M9
2	RNA-seq	chrL	BPNC10044R	D286.1_srna_0171	592835	593164	Hypothetical protein	D286.1_0591	BPSL0592	
3	RNA-seq	chrL	BPNC10089F	D286.1_srna_0415	1393459	1393623	Hypothetical protein	D286.1_1195	BPSL1302	
4	RNA-seq	chrL	BPNC10110F_308_SIPHT	D286.1_srna_0578	2102282	2102535	Phb polymerase	D286.1_1761	BPSL1622	
							Exported protein	D286.1_1762	BPSL1623	
5	RNA-seq	chrL	BPNC10163R_424_SIPHT	D286.1_srna_0759	2695220	2695483	Peptidase C26	D286.1_2264	BPSL2337	
6	RNA-seq	chrL	BPNC10187R	D286.1_srna_0890	3099055	3099630	Lipoprotein	D286.1_2621	BPSL2034	
7	RNA-seq	chrL	BPNC10146F	D286.1_srna_0696	2493413	2493667	Acyl binding family protein	D286.1_2112	BPSL2186	Gene UP and sRNA DOWN in M9
8	RNA-seq	chrL	BPNC10134F	D286.1_srna_0462	1589591	1589896	H-ns histone family protein	D286.1_1369	BPSL2034	Gene DOWN and sRNA DOWN in M9
9	RNA-seq	chrL	Candidate_820_SIPHT	D286.1_srna_0533	1811088	1811441	tRNA dihydrouridine synthase a family	D286.1_1553	BPSL1852	
10	RNA-seq	chrL	BPNC10201R	D286.1_srna_0980	3427831	3428197	Novel gene 8a	D286.1_2913	na	
11	RNA-seq	chrL	BPNC10146F	D286.1_srna_0696	2493413	2493667	Dead deah box helicase family protein	D286.1_2113	BPSL2187	
12	RNA-seq	chrL	Candidate_1127_SIPHT	D286.1_srna_1045	3703461	3703621	Ribosomal proteins S10	D286.1_3174	BPSL3214	
13	RNA-seq	chrL	BPNC10146R	D286.1_srna_0692	2459242	2459430	Ribosomal protein S2	D286.1_2084	BPSL2159	
14	RNA-seq	chrL	BPNC10209R	D286.1_srna_1051	3723032	3723160	Ribosomal protein S10	D286.1_3187	BPSL3223	
			BPNC10197F	D286.1_srna_1052	3723032	3723160	Ribosomal protein 1	D286.1_3188	BPSL3224	
15	RNA-seq	chrL	BPNC10196F_1129_SIPHT	D286.1_srna_1049	3721745	3721913	Ribosomal protein 7	D286.1_3186	BPSL3222	
16	RNA-seq	chrL	Candidate_29_SIPHT	D286.1_srna_1104/ D286.1_srna_1105	3896200	3896553	Glycine cleavage system t protein	D286.1_3333	BPSL3360	
17	RNA-seq	chrS	BPNC2008R_567_SIPHT	D286.2_srna_0021	73653	74208	Hypothetical protein	D286.2_0069	antisense but not in k96243	Gene UP sRNA UP in M9
18	RNA-seq	chrS	BPNC20025F	D286.2_srna_0106	427693	428100	Taurine catabolisme dioxygenase	D286.2_0330	BPSS0309	
19	RNA-seq	chrS	BPNC20037R	D286.2_srna_0137	567734	568027	Hypothetical protein	D286.2_0435	na	
							Polysaccharide-biosynthesis	D286.2_0436	BPSS0417	
20	RNA-seq	chrS	BPNC20128R	D286.2_srna_0476	2218835	2219131	Diaminobutyrate	D286.2_1696	BPSS1656	
							Thiamine-pyrophosphate	D286.2_1697	BPSS1657	
21	RNA-seq	chrS	BPNC20147R_281_SIPHT	D286.2_srna_0560	2562887	2563316	Metallo-beta-lactamase	D286.2_1969	BPSS1916	
							Acetoacetyl-reductase	D286.2_1970	BPSS1917	
22	RNA-seq	chrS	BPNC20086R_360_SIPHT	D286.2_srna_0328	1409405	1409721	Exported protein	D286.2_1086	BPSS1038	Gene DOWN sRNA DOWN in M9

23	sRNA-seq	chrL	Candidate_644_SIPHT	D286.1_srna_0312	1075881	1075942	Gram-negative porin family protein	D286.1_0932	BPSL1029	Gene UP sRNA UP in M9
24	sRNA-seq	chrL	Candidate_199_SIPHT	D286.1_srna_0932	3269232	3269349	Glycine zipper family protein	D286.1_0946	BPSL1043	
25	sRNA-seq	chrL	BPNC10113R	D286.1_srna_0567	1986019	1986182	Mbth-like family protein	D286.1_1689	BPSL1726	
26	sRNA-seq	chrL	BPNC10110F_308_SIPHT	D286.1_srna_0578	2102282	2102535	Phb depolymerase family protein	D286.1_1761	BPSL1623	
							Exported protein	D286.1_1762	BPSL1622	
27	sRNA-seq	chrL	Candidate_581_SIPHT	D286.1_srna_1107	3911718	3911970	Acetyldehyde dehydrogenase	D286.1_3342	BPSL3369	
28	sRNA-seq	chrL	BPNC10233R_28_SIPHT	D286.1_srna_1122	3972025	3972210	ABC transporter family protein	D286.1_3391	BPSL3417	
29	sRNA-seq	chrL	BPNC10048R	D286.1_srna_0193	695212	695428	Phoh-like family protein	D286.1_0613	BPSL0673	Gene DOWN sRNA DOWN in M9
30	sRNA-seq	chrL	Candidate_825_SIPHT	D286.1_srna_0523	1789117	1789269	Methyl accepting chemotaxis signaling domain protein	D286.1_1500	BPSL1905	
31	sRNA-seq	chrL	BPNC10146R	D286.1_srna_0692	2459242	2459430	Ribosomal protein s2	D286.1_2084	BPSL2159	
32	sRNA-seq	chrL	BPNC10209R	D286.1_srna_1051	3723032	3723160	Ribosomal protein S10	D286.1_3187	BPSL3223	
33	sRNA-seq	chrL	Candidate_1127_SIPHT	D286.1_srna_1045	3703461	3703621	Ribosomal proteins S10	D286.1_3174	BPSL3214	
34	sRNA-seq	chrL	Candidate_1133_SIPHT	D286.1_srna_1063	3751416	3751532	NADP-dependent malic enzyme	D286.1_3208	BPSL3242	
35	sRNA-seq	chrL	Candidate_1158_SIPHT	D286.1_srna_1101	3873620	3873896	Lipoprotein	D286.1_3313	BPSL3336	
36	sRNA-seq	chrL	BPNC20048F	D286.1_srna_1106	3898521	3898678	Glycine dehydrogenase	D286.1_3335	BPSL3362	
37	sRNA-seq	chrS	BPNC2008R_567_SIPHT	D286.1_srna_0021	73653	74208	Hypothetical protein	D286.2_0069	na	Gene UP sRNA UP in M9
38	sRNA-seq	chrS	Candidate_172_SIPHT	D286.2_srna_0280	1168878	1169001	Cbs domain protein	D286.2_0917	BPSS0882	
39	sRNA-seq	chrS	Candidate_345_SIPHT	D286.2_srna_0286	1202112	1202195	Multispp:hypothetical protein	D286.2_0949	BPSS0914	
40	sRNA-seq	chrS	Candidate_369_SIPHT	D286.2_srna_0355	1552528	1552581	Cytc3	D286.2_1193	BPSS1183	
41	sRNA-seq	chrS	Candidate_400_SIPHT	D286.2_srna_0434	1938096	1938248	Biotin carboxyl	D286.2_1478	BPSS1445	
							Enoyl hydratase isomerase family protein	D286.2_1479	BPSS1446	
42	sRNA-seq	chrS	Candidate_239_SIPHT	D286.2_srna_0463	2083884	2084038	Serine carboxy peptidase family protein	D286.2_1596	BPSS1561	
43	sRNA-seq	chrS	Candidate_244_SIPHT	D286.2_srna_0475	2218652	2218813	Diaminobutyrate 4-transaminase family protein	D286.2_1675	BPSS1635	
							Thiamine pyrophosphate central domain protein	D286.2_1676	BPSS1636	
44	sRNA-seq	chrS	Candidate_468_SIPHT	D286.2_srna_0575	2606161	2606374	Poly-beta-hydroxybutyrate polymerase family protein	D286.2_2008	BPSS1954	
45	sRNA-seq	chrS	BPNC20163F	D286.2_srna_0591	2673939	2674307	Multispp:hypothetical protein	D286.2_2058	BPSS2001	
46	sRNA-seq	chrS	Candidate_316_SIPHT	D286.2_srna_0668	3020302	3020536	HSP20 alpha crstallin family protein	D286.2_2340	BPSS2288	
47	sRNA-seq	chrS	BPNC20163R	D286.2_srna_0615	2833443	2833586	Multispp:hypothetical protein	D286.2_2177	BPSS2129	Gene DOWN and sRNA DOWN in M9

Supplementary Table S6 – Interaction of sRNAs with their target genes predicted by CopraRNA

*Provided in Supplementary_Table_S6.xls (the interaction of sRNAs with their flanking genes were coloured by red while the others showed the sRNA's interaction with distantly encoded genes)

Sheet 1: (A) BPNC10044R
The putative interactions between BPNC10044R sRNA and its predicted target genes.

Sheet 2: (B) Candidate_369_SIPHT
The putative interactions between Candidate_369_SIPHT sRNA and its predicted target genes.

Sheet 3: (C) BPNC10048R
The putative interactions between BPNC10048R sRNA and its predicted target genes.

Sheet 4: (D) BPNC10196F_1129_SIPHT
The putative interactions between BPNC10196F_1129_SIPHT sRNA and its predicted target genes.

Sheet 5: (E) BPNC10209R
The putative interactions between BPNC10209R sRNA and its predicted target genes.

Sheet 6: (F) BPNC10146R
The putative interactions between BPNC10146R sRNA and its predicted target genes.

Sheet 7: (G) BPNC10037R
The putative interactions between BPNC10037R sRNA and its predicted target genes.

Sheet 8: (H) BPNC10233R_28_SIPHT
The putative interactions between BPNC10233R_28_SIPHT sRNA and its predicted target genes.

Sheet 9: (I) Candidate_468_SIPHT

The putative interactions between Candidate_468_SIPHT sRNA and its predicted target genes.

Sheet 10: (J) BPNC10113R

The putative interactions between BPNC10113R sRNA and its predicted target genes.

Sheet 11: (K) BPNC10134F

The putative interactions between BPNC10134F sRNA and its predicted target genes.

Sheet 12: (L) BPNC10146F

The putative interactions between BPNC10146F sRNA and its predicted target genes.

Supplementary Table S7 – List of designated primers for the amplification of ten sRNA candidates together with eleven flanking genes and reference gene of *B. pseudomallei* D286 for both standard PCR and qRT-PCR assays

Table S7. A) Primers of target sRNAs used in this study

sRNA ID (Bp D286.1_srna_*, BPNC*)	Primers	Sequences	GC% and Tm	Product size (bp)
D286.1_srna_0171, BPNC10044R	0171_F<br>0171_R	GTAGAGCAAGCCACGTTCC<br>GAAAACCGGCGTGTCGAG	%GC: 58 and Tm: 64°C<br>%GC: 61 and Tm: 65°C	280
D286.1_srna_0567, BPNC10113R	0567_F<br>0567_R	GCGTGCCTTGAAGATGAGAG<br>ATGTACCGCCTTGGCATCG	%GC: 55 and Tm: 64°C<br>%GC: 58 and Tm: 66°C	164
D286.1_srna_0193, BPNC10048R	0193_F<br>0193_R	AGCCGTCGAGCCGTC<br>ACGGCGGTGCGCAAG	%GC: 73 and Tm: 65°C<br>%GC: 80 and Tm: 68°C	172
D286.1_srna_1049, BPNC10196F_1129_SIPHT	1049_F<br>1049_R	ATGGCCGTCCGACGGCA<br>AACCGCCCATCCCTCGACG	%GC: 71 and Tm: 60<br>%GC: 68 and Tm: 64	164
D286.1_srna_1051, BPNC10209R	1051_F<br>1051_R	GGGAAAGCAGCAGTCGG<br>CTCGTTAGGAGTATTTCCGCTG	%GC: 65 and Tm: 64°C<br>%GC: 50 and Tm: 64°C	129
D286.1_srna_0692, BPNC10146R	0692_F<br>0692_R	CTTTGGCTAAGTCGACGCTG<br>GGCCCTCGAAAATTGAAACC	%GC: 55 and Tm: 64°C<br>%GC: 50 and Tm: 63°C	144
D286.1_srna_0144, BPNC10037R	0144_F<br>0144_R	GCGCGTTATCGACATTTCCG<br>AACGAAGGTTCAGAGCCCG	%GC: 55 and Tm: 60°C<br>%GC: 58 and Tm: 65°C	206
D286.1_srna_1122, BPNC10233R_28_SIPHT	1122_F<br>1122_R	GCGCGAACCGAACGC<br>GGCCGGTCTCCTCGC	%GC: 73 and Tm: 65°C<br>%GC: 80 and Tm: 65°C	183
D286.1_srna_0462, BPNC10134F	0462_F<br>0462_R	TGAAGATGATCCGAGCCCTTG<br>TTGGATGAAAGACGGCAAGC	%GC: 52 and Tm: 65°C<br>%GC: 50 and Tm: 64°C	282
D286.1_srna_0696, BPNC10146F	0696_F<br>0696_R	GGCGCAGCAAAAACCG<br>GACGTGCGGACAACAATTTC	%GC: 63 and Tm: 63°C<br>%GC: 50 and Tm: 63°C	232

Table S7. B) Primers of target genes used in this study

gene ID (BPSL* or BPSS*, Bp D286.1_*), protein ID	Genomic coordinates, strand position in Bp D286	Primers	Sequence	Product size (bp)
bpsl0591<br>D286.1_0530 , WP_004522838	589463:592729, reverse	D286.1_0530_F<br>D286.1_0530_R	TCAAGACGATCCTCTACGCG<br>TGCAGATAGTAGGTCTCGGC	187
bpsl1725<br>D286.1_1688 , WP_004544517	1983429:1985333, reverse	D286.1_1688_F<br>D286.1_1688_R	CTGTTGAATCGTGCGGAGG<br>GCAAATCGTCTCGTGGTCC	177
bpsl0672<br>D286.1_0612, YP_107301	694130:694942, reverse	D286.1_0612_F<br>D286.1_0612_R	GTCGTCGAAAAGGAAGCGC<br>AGCGGTATGGATTCGGGAAG	184
bpsl3221<br>D286.1_3185, YP_109815	3717559:3721665, reverse	D286.1_3185_F<br>D286.1_3185_R	CATCAATCAGCGTCCGATCG<br>CGTTCAGCTCTTCGATGTGG	221
bpsl3223<br>D286.1_3187, YP_109817	3722467:3722964, reverse	D286.1_3187_F<br>D286.1_3187_R	GAGAAGACAAGCAAGCCGTC<br>TCCGAGATGCCGTAGATCAG	244
bpsl2159<br>D286.1_2084, YP_108754	2458419:2459159, reverse	D286.1_2084_F<br>D286.1_2084_R	TCCCTTCATCTTCGGTCACC<br>TTGAAGTTGGTCAGCATGCC	231
bpsl0493<br>D286.1_0466, YP_001064832	509014:510780, reverse	D286.1_0466_F<br>D286.1_0466_R	ATCGGTGATGCAGGGCTATC<br>ATGGCTGTCGATCGTCTCC	199
bpsl0494<br>D286.1_0467, YP_006651337	511214:512137, forward	D286.1_0467_F<br>D286.1_0467_R	AGCATCAAGGAGGGGCTG<br>GGAAACAGCACGAACGTCTC	188
bpsl3418<br>D286.1_3393, YP_001068288	3972420:3974105, reverse	D286.1_3393_F<br>D286.1_3393_R	CTCGAATATCACGTGCAGCC<br>TCGGTCGACAGATAGTTCGG	176
bpsl2035<br>D286.1_1369, YP_108632.1	1589246:1589527, reverse	BPSL2035_F<br>BPSL2035_R	GGAAACCTACCTCGACCTTC<br>GACGATTCGCTTCACTTCGG	109
bpsl2187<br>D286.1_2113, YP_001066782	2493758:2495299, forward	D286.1_2113_F<br>D286.1_2113_R	CCGAACTGAAGATGCTCGTG<br>CTTCTGCGTGATCTCGATGC	197
bpsl2827<br>dnaK	3380019:3381971, reverse	dnaK_F<br>dnaK_R	TTCGAAGTGCTGTCGACCAA<br>TTGATTTCGGTCTGCTGGCT	200
bpsl0501<br>cydB	550571:551707, reverse	cydB_F<br>cydB_R	TTCGAAGTGCTGTCGACCAA<br>TTGATTTCGGTCTGCTGGCT	230
bpsl1518<br>hfq	1757798:1758037, forward	bpsl1518_F<br>bpsl1518_R	CAAGACCCGTTTTTGAACGC<br>CGTCGAAATGGCGTGCTTG	162

Supplementary Figure 8 – Semi-quantitative detection of ten sRNA candidates together with eleven flanking genes of *B. pseudomallei* D286 in BHIB vs M9 media using end-point RT-PCR assays

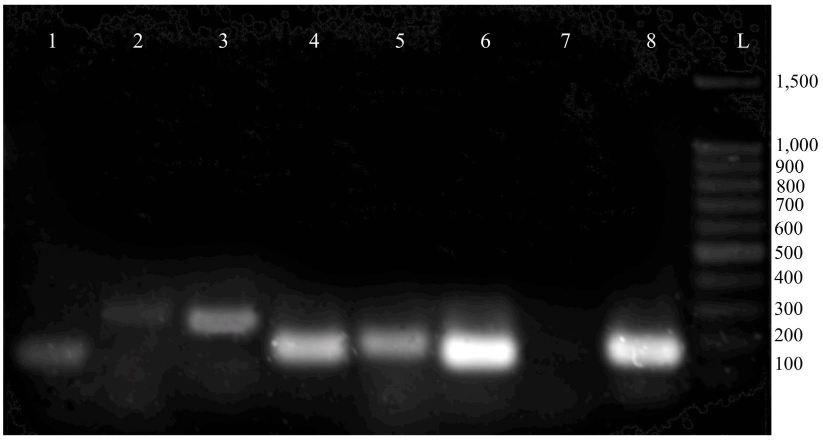

1 - BPNC20021R (160 bp)
2 - BPNC10044R (280 bp)
3 - BPNC10037R (206 bp)
4 - BPNC10113R (164 bp)
5 - BPNC10233R_28_SIPHT (183 bp)
6 - *bpsl1518* (+)ve (162 bp)
7 - RC (-)ve
8 - *dnaK* (181 bp)
L - 100 bp DNA ladder

A) PCR analysis of BPNC20021R, BPNC10044R, BPNC10037R, BPNC10113R and BPNC10233R_28_SIPHT sRNAs using standardly prepared cDNA samples of *B. pseudomallei* D286 grown under BHIB/M9 condition for the purpose of primer validation prior to qPCR assay. The remaining positive (*bpsl1518* and *dnaK* genes) and negative (RC) controls were included in the respective lanes.

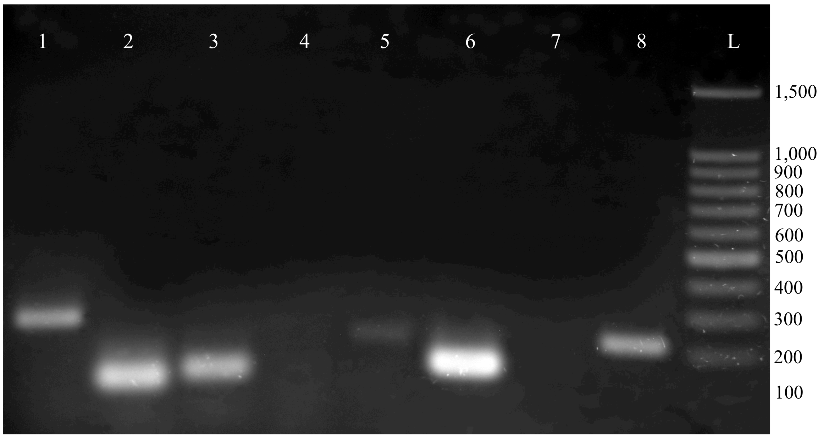

1 - BPNC10134F (282 bp)
2 - BPNC10209R (129 bp)
3 - BPNC10146R (144 bp)
4 - BPNC10048R (n/a)
5 - BPNC10146F (232 bp)
6 - *bpsl1518* (+)ve (162 bp)
7 - RC (-)ve
8 - *cydB* (+)ve (230 bp)
L - 100 bp DNA ladder

B) PCR analysis of BPNC10134F, BPNC10209R, BPNC10146R, BPNC10048R and BPNC10146F sRNAs using standardly prepared cDNA samples of *B. pseudomallei* D286 grown under BHIB/M9 condition for the purpose of primer validation prior to qPCR assay. The remaining positive (*bpsl1518* and *cydB* genes) and negative (RC) controls were included in the respective lanes.

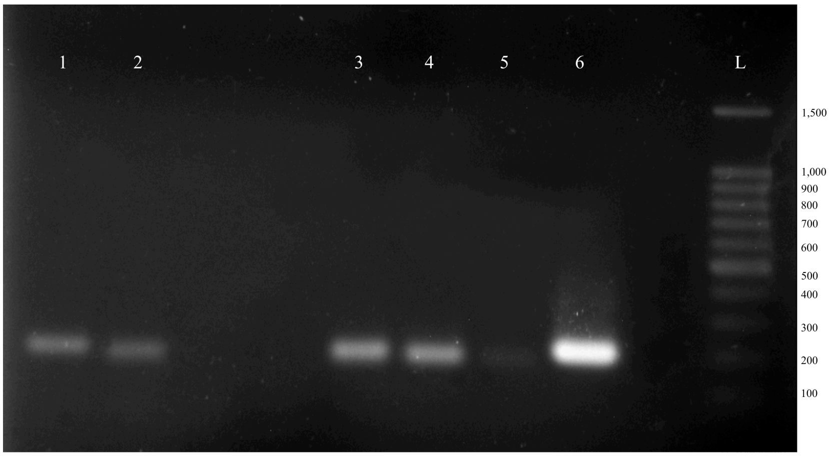

Lane number:

1 - *bpsl0493* (199 bp)

2 - *bpsl0494* (188 bp)

3 - *bpsl0672* (184 bp)

4 - *bpsl1725* (177 bp)

5 - *bpsl2035* (173 bp)

6 - *bpsl2187* (197 bp)

L - 100 bp DNA ladder

C) PCR analysis of six flanking genes, *bpsl0493*, *bpsl0494*, *bpsl0672*, *bpsl1725*, *bpsl2035* and *bpsl2187* using standardly prepared cDNA samples of *B. pseudomallei* D286 grown under BHIB/M9 condition for the purpose of primer validation prior to qPCR assay.

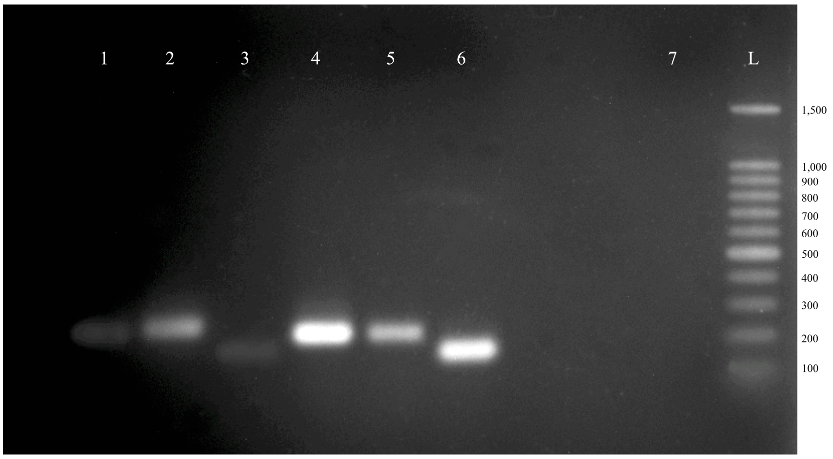

Lane number:

1 - *bpsl2159* (231 bp)

2 - *bpsl3223* (244 bp)

3 - *bpsl3418* (176 bp)

4 - *bpsl3221* (221 bp)

5 - *bpss0191* (239 bp)

6 - BPNC10196F_1129_SIPHT (164 bp)

7 - RC (-)ve

L - 100 bp DNA ladder

D) PCR analysis of five flanking genes, *bpsl2159*, *bpsl3223*, *bpsl3418*, *bpsl3221*, *bpss0191* and BPNC10196F_1129_SIPHT sRNA using standardly prepared cDNA samples of *B. pseudomallei* D286 grown under BHIB/M9 condition for the purpose of primer validation prior to qPCR assay. The negative control (RC) was included in the lane numbered 7.

Supplementary Table 9 – CopraRNA analysis of RNA-RNA interaction between *cis*-sRNA and flanking genes

*Provided in Supplementary_Table_S9.xls

Sheet 1: RNA-RNA interaction

CopraRNA analyses of RNA-RNA interactions between cis-sRNA and flanking genes.
